# The Use of Ceramics as Bone Substitutes in Revision Hip Arthroplasty

**DOI:** 10.3390/ma2041895

**Published:** 2009-11-19

**Authors:** Michael R. Whitehouse, Ashley W. Blom

**Affiliations:** 1 Department of Academic Orthopaedic Surgery (University of Bristol), BIRC, Lower Level AOC, Southmead Hospital, Westbury-on-Trym, Bristol, BS10 5NB, UK; E-Mail: ashley.blom@nbt.nhs.uk (A.W.B.); 2 Avon Orthopaedic Centre, Southmead Hospital, Westbury-on-Trym, Bristol, BS10 5NB, UK

**Keywords:** ceramics, grafting, bone, hip arthroplasty, revision

## Abstract

The number of grafting procedures, including those performed in primary and revision hip arthroplasty, continues to rise around the world. Demand for musculoskeletal donor tissue now outstrips supply. There is no single bone substitute that is ideal for all circumstances. Bone substitutes act as a scaffold and are usually osteoconductive. They are rarely osteoinductive; if they are, a molecular bond is formed between the graft and host bone, improving fixation and longevity. Bone graft substitutes are very rarely osteogenic. There is a growing body of clinical evidence supporting the use of bone graft substitutes *in vivo* for complex hip arthroplasty.

## 1. Introduction

One of the greatest challenges facing the orthopaedic surgeon in revision arthroplasty is the restoration of lost bone stock. Aseptic loosening remains the primary failure mode for cemented and uncemented hip replacements. Wear particles released from the implanted materials provoke an inflammatory host response, stimulating osteoclasts to resorb bone [1,2].

Bone has a complex structural hierarchy with the ability to adapt its structure to suit the mechanical loads to which it is exposed [3]. Cortical and cancellous bone exhibits different mechanical properties due to their different porosities. Bone constitutes approximately 45% bone mineral which approximates to hydroxyapatite (Ca_10_(PO_4_)_6_(OH)_2_) [4]. *In vivo*, bone behaves as a two-phase composite of a collagen matrix reinforced by hydroxyapatite [5].

Autografting is considered to be the gold standard for cases requiring restoration of bone stock. There are substantial limitations to the technique that make it impractical in the majority of revision hip procedures. These include bulk limitations, high rates of graft donor site morbidity [6] and increased duration of the procedure. These limitations have led to an increase in the use of allograft. Impaction grafting of both the acetabulum and femur in revision surgery was reported by the Nijmegen and Exeter groups in the early 1990s [7,8,9]. The technique of impaction grafting of either the femoral or acetabular side involves the creation of a contained defect. This containment is achieved with the use of mesh, screws, bulk graft or other augments. Once containment has been achieved, the graft of choice is pushed into the defect before being impacted into place by serial tamps. These tamps are gradually increased in size in order to achieve effective impaction of the graft and to provide a stable bed for subsequent implantation of the prosthesis with or without cement. Allograft bone is not reproducible and varies according to donor factors (donor age, bone mineralisation) and processing (sterilisation and storage techniques). Mixed results of impaction grafting have been reported [7,10,11,12,13] but these have improved in non-originator centres over time. Infection remains a concern [14,15].

More than 500,000 bone grafting procedures are performed annually in the USA [16]. Projected donation rates are not sufficient to meet this demand. It was predicted in 1998 that demand would outstrip supply for allograft in Scotland by 2000 due to an increase in the revision burden of 100% between 1995 and 2000 [17]. This shortfall in supply and concerns regarding results has necessitated the development of alternative bone substitutes.

Investigated alternatives include:

1. Xenografts
2. Corraline derived hydroxyapatite
3. Polymethylmethacrylate
4. Calcium sulphate
5. Polyhydroxyacids
6. Glass-ionomer ceramics
7. Absorbable ceramics
8. Collagen matrix

A number of these materials have limitations, particularly in the context of impaction grafting in revision surgery [18]. Impaction grafting involves high compressive forces; collagen matrices and polyhydroxyacids lack the requisite strength to be used in the context of such forces. Xenografts and coralline grafts both provoke a host response and have the potential to transmit infection [19,20]. Xenografts do not osseointegrate as well as allograft and demonstrate the same variability in particle size, morphology and mechanical properties as allograft. Calcium sulphate (plaster of Paris) is resorbed too rapidly [21] and the results of promising series have not been replicated [22]. Glass ionomer and polymethylmethacrylate are not resorbed and therefore cannot be replaced by host bone.

As described by Blom *et al.* [18], an ideal bone graft substitute should impart structural stability, allow neo-ossification by means of osseoconduction, osseoinduction and substitution, be cost effective, available in unlimited supply and have no potential for transmission of infection or provocation of a detrimental host response. Bone is considered a primary model for tissue engineering in that a scaffold alone may be implanted with recruitment of the necessary cells and components from the host tissue [4]. This review will focus on the use of ceramics as bone substitutes.

## 2. Ceramics

### 2.1. Ceramics

Ceramics are inorganic, non-metallic solids. Sintering is an important part of the preparation process of the ceramics. This involves heating the ceramic to a temperature below the melting point. During the sintering process, the density of the ceramic increases in relation to a reduction in the porosity of the ceramic. It is during the sintering process that the polycrystalline nature of the final ceramic is formed. The grain size distribution formed during this stage of the processing is an important determinant of the final mechanical properties. A temperature of 900 °C is required to form grain boundaries for biphasic calcium phosphate ceramics; the grain boundaries also play an important role in determining the final mechanical properties [23].

### 2.2. Glass-Ionomer Ceramics

The common property of the glass-ionomer ceramics is the presence of glass. They are manufactured by sintering the glass in different proportions of SiO_2_, Al_2_O_3_, CaF_2_ and AlPO_4_ with or without the addition of hydroxyapatite. As stated earlier, they are not resorbable; this is due to the presence of the silicate and aluminium. The non-porous nature of the glass ionomers means that they only allow peripheral osseointegration with no osseoconduction within the particles. They do have the advantage of not promoting foreign body reactions when implanted [24]. Although it is clear that the glass-ionomer becomes incorporated into the bone matrix, it is unclear whether this is detrimental or perhaps even beneficial.

### 2.3. Absorbable Ceramics

Synthetic hydroxyapatite (Ca_10_(PO_4_)_6_(OH)_2_), tricalcium phosphate (Ca_3_(PO_4_)_2_) and combinations of the two are commonly used as bone graft substitutes [25]. These are formed by the precipitation of a powder from an aqueous solution at a certain pH range. The powder is then cold pressed to form tablets and these tablets are then sintered to produce a material with porosity by volume in the range of 1 to 5%. Porosity to allow osseoconduction is achieved by the addition of materials that create porosity within the structure and are then burnt off during the sintering. Examples include glucose and naphthalene. An alternative to these techniques is the preparation of commercially available porous hydroxyapatite (HA) from natural cancellous bone. Mechanical and structural properties have been observed to vary widely amongst specimens of these substances [26]. Densities of Endobon® specimens range from 0.35 to 1.44 g·cm^-3^, and across this range of densities the ultimate compressive stress was seen to increase from 1 to 11MPa and the ultimate compressive modulus from 0.2 to 3.1 GPa. Isotropic specimens were also observed to poses higher compressive moduli than anisotropic specimens of the same density.

HA and tricalcium phosphate (TCP) have been shown to osseointegrate [27,28]. The coating of human bone marrow stromal cells (HBMSC) with amino-acid functionalized HA nanoparticles leads to a significant increase in osteoblast differentiation when compared to uncoated cells [29]. The *in vivo* component of this study demonstrated comparable osteoid formation after 21 days in immunocompromised mice. The seeding of poly(DL-lactic acid) (PLA) graft with HBMSCs leads to improved results *in vitro* and *in vivo* animal models when compared to PLA alone [30]. *In vitro* testing demonstrated improved results in shear testing and *in vivo* testing revealed a significant increase in new bone and blood vessel formation. Green *et al*. have demonstrated that porous calcium carbonate microspheres confined to a 3D pellet promote *in vitro* formation of osteoid tissue [31] and that these microspheres can augment *in vivo* bone formation when used in impaction grafting [32].

HA and TCP do have problems with maintaining structural integrity under load [33]. A pore size of 300-500 μm has been shown to be optimal for osseoconduction [34,35]. Bouler *et al*. demonstrated that the thickness of the bridges between the pores were important; when these fell below a critical size, the ceramic disintegrated when exposed to low compressive forces [23].

Until recently it was suggested that the mechanical properties of the calcium phosphate materials were not sufficient to permit their use where they must bear the initial structural load alone [33]. This would not appear to be the case. In its non-porous state, the tensile and compressive strength is far in excess of that of cancellous and cortical bone. In its porous state, the compressive strength is equal to that of cancellous bone and the tensile strength 72% that of cancellous bone [36]. Plugs of HA/TCP granules and HA/TCP combined with a collagen matrix demonstrate no significant difference to autologous graft, allograft cortical bone or host bone following 6 months of ad lib weight bearing in canine tibia when peak torque, stiffness and energy to peak torque were analysed [37]. The difficulty faced with the application of bioactive ceramics to the *in vivo* environment is in the departure of their mechanical behaviour from that of human cancellous or cortical bone. The ceramics are more brittle, their fracture toughness is lower and the elastic modulus higher for the bioactive ceramics, even in the case of glass-ceramic A-W which exhibits higher mechanical strength than the other bioactive ceramics [38]. Bone is a composite structure consisting of nano-scale apatite crystals deposited onto a 3 dimensional collagen matrix. It is the composite structure, which gives bone its inherent mechanical properties, and attempts to simulate this structure can lead to improved graft mechanical properties. Combination of ceramic with a polymer such as cellulose, leads to improved bending strength and Young’s modulus [39]. A significant deterioration in these properties was observed over 14 days with soaking in simulated body fluid. It was felt this decline was due to the degradation of the adhesion between the ceramic particles and cellulose fibres. An alternative approach is to embed to ceramic in a self-hardening carrier such as fibrin glue, this allows the customised shaping of the graft and within six weeks, the mechanical properties of the composite are approaching that of the trabecular bone [40].

Bouler *et al*. studied the influence of synthesis parameters on the compressive strength of porous biphasic calcium phosphate ceramics [23]. Two different ratios of HA:TCP were studied, 45:55 and 75:25, the former demonstrated better compressive strengths. Other variables analysed included percentage and mean size of macropores, sintering temperature and isostatic compression pressure. Of these the isostatic compression pressure was the least important. At set volume percentage porosity, a smaller number of pores of approximately 500 μm were better than many of 100 μm. The thickness of the bridges between the pores was critical, when the size of these fell below a critical limit the compressive strength was severely compromised. The porosities within the structure must communicate in order to allow cellular ingress into the structure; the porosity however needs to remain above a certain threshold for that application in order to provide the necessary mechanical support to facilitate the process [41]. The authors felt that the mechanical properties of the scaffold structure of the graft were an important consideration when comparing 70% total porosity specimens to 80%. The percentage strut porosity of the graft was related to the bone volume formed at three weeks and the mineral apposition rate at one to two weeks; with 20% strut porosity associated with higher volumes and rates than 10%.

The porosity of the ceramic also influences the rate of resorption of the ceramic due to the increase in surface area. Two processes dictate the rate of resorption: dissolution and phagocytosis. These have been observed in calcium phosphate ceramics that has been implanted into ovine bone [42]. The process appeared to occur by a variety of mechanisms; multinucleated giant cells causing localised areas of resorption and a generalised uniform dissolution at the implant surface. HA and TCP have been observed to resorb at different rates. When rates of dissolution were compared in buffered acid and base solutions, the TCP dissolved 12 times faster in acid solution and 22 times faster in base solution [36]. HA and TCP ceramics have also been observed to resorb at different rates when implanted into rabbit bone. Over 24 weeks over 46% of the TCP resorbed compared to over 27% of the HA yet the HA allowed 8% more new bone formation [43]. The rate of resorption of carbonate-substituted HA appears to related to the carbonate content with increased resorption with increasing carbonate content up to 2.35 wt% [44].

The structure of the ceramic utilised can be manipulated in an attempt to increase the biological response of the host tissue. The aim of these manipulations is to decrease the period of time taken for the construct to achieve the mechanical strength to bear load and therefore aid patient rehabilitation. It has been suggested that the use of stoichiometric hydroxyapatite leads to a faster biological response than commercial hydroxyapatite [4]. In simulated body fluid tests, the time until production of an apatite layer was reduced by 30% compared to stoichiometric hydroxyapatite.

The use of Bioglass® in conjunction with a poly(DL-lactic acid) matrix had an inverse dose related effect on osteoblast activity if not pre-treated; if the scaffold was pre-treated with serum for 24 hours, 5 wt% Bioglass® composite was associated with an increase in alkaline phosphatase activity when compared to 0 wt% and 40 wt% samples [45].

Bone coverage and ingrowth has been observed to occur more rapidly in some bioactive glasses and glass ceramics than in hydroxyapatite [46,47]. Substitution of ions including sodium, magnesium and silicon is of interest as these ions are present *in vivo* and may improve the coverage and ingrowth rates. Ionic substitution affects the apatite properties such as lattice parameters, crystal size and crystallinity. These in turn influence the stability and solubility of the HA formed. Silicon is of particular interest as it has been identified at around 5 wt% at sites of active calcification [48]. *In vitro* studies have demonstrated that physiological levels of silicon stimulate osteoblastic cell differentiation and type 1 collagen synthesis [49]. It is possible to incorporate silicon (Si) into HA by direct substitution for phosphorus (or phosphate). Up to 0.4 wt% silicon substitution can be achieved by a simple aqueous precipitation method [50], and levels of up to 1.5 wt% can be substituted as a single phase [4,50]. The amount of bone ingrowth and coverage in an animal *in vivo* model is significantly improved in the case of both 0.8 wt% and 1.5 wt% silicate-substituted hydroxyapatite when compared to pure hydroxyapatite [51]. Investigations of the mechanism behind this indicate that increased dissolution at grain boundaries and triple junctions may be responsible for the increased rate of bone ingrowth and coverage observed [52]. At six weeks *in vivo*, organised ingrowth of collagen fibrils can be observed at the surface of SiHA, deeper within the implant poorly organised and mineralised fibrils are observed [53]. Titanium substitution has been recently investigated [54]. HA containing levels of up to 1.6 wt% by co-precipitation were produced and investigated following *in vitro* culture with primary human osteoblasts. Significantly higher cellular activity was observed for 0.8 wt% TiHA than with HA and this was associated with well-organised actin cytoskeletal protein formation after 1 day in culture. This holds promise for future investigation.

The method of preparation of HA/TCP can have an effect on the subsequent biological activity associated with the particles *in vitro*. A sintering temperature of 110 °C stimulated release of interleukin (IL)-1β, IL-6, tumour necrosis factor (TNF) α, prostaglandin (PG)E2 whereas particles sintered at 900 and 1,200 °C did not [55]. Particles that had been plasma sprayed also failed to release inflammatory mediators. The addition of HA particles to cell culture causes an initial significant reduction in cell counts [56]. The subsequent effect on the cell counts, mediated by TGF-β1 and PGE2, are dependent upon the particle size in the culture. Particles in the 37 to 63 and 177 to 250 μm ranges were associated with a more rapid increase in cell counts over seven days than were particles in the 0.5 to 30 or 420 to 841 μm ranges.

Comparative studies of ceramic bone graft substitutes have revealed some differences between different compositions in terms of their *in vivo* animal model performance. A comparison of four different porous biphasic calcium phosphate ceramics (HA-βTCP), one of which was reinforced with a bioresorbable polylactic acid, a pure HA ceramic and a carbonated apatite (CA) ceramic revealed that neither the HA or CA ceramic were osteoinductive in soft tissue and had inferior bone integration when compared to the HA-βTCP. The addition of the polylactic acid reduced osseoinduction and integration [57]. A comparative study of dense calcium sulphate, βTCP and a porous silicated calcium phosphate over 12 weeks revealed that the former underwent rapid dissolution and elicited a mild inflammatory response and the latter two supported early bone apposition [58]. The βTCP samples then underwent an inflammatory response impairing further bone deposition and leading to bone resorption, which the silicated samples did not.

It has been established that the synthesis parameters of the ceramic play a vital role in its success *in vivo* and that these parameters may be common to different ceramic compositions. Porous biphasic ceramics perform well *in vivo* and carbonation and the addition of polylactic acid are not beneficial. The substitution of both silicon and titanium into the ceramic structure hold promise but we have yet to determine the success of such techniques in human *in vivo* studies and what the ideal synthesis parameters and composition may be for these ceramics. Delivery of the ceramic either as a pre-formed composite or in a self-hardening composite solution may prove beneficial if further work can improve the long-term adhesion between the ceramic and polymer components. Ceramic bone substitutes have an effect on the cellular environment when implanted and synthesis parameters again appear to influence this. The future direction of ceramic bone substitute development will focus on the refining the composition and synthesis parameters of the ceramics and their interaction with the cellular environment and how we may best influence this to improve the integration and survival of the graft.

### 2.4. Clinical Use of Ceramics

The first series reporting the use of ceramics (TCP) as a bone graft substitute appeared in 1988. The results in 43 trauma patients were reported and although the follow up was short and incomplete, the initial results were promising [59]. The first report of hydroxyapatite ceramic use as a bone graft substitute in revision arthroplasty surgery appeared as a small part of a series of 45 cases reported in 1990 [60]. Porous biphasic ceramics have been shown to be a successful and safe material for use in impaction grafting in an ovine model, with clinical, radiological and histological changes comparable to allograft [61]. This material has been used successfully in an unselected consecutive cohort of patients undergoing acetabular reconstruction during revision hip surgery [62].

Oonishi *et al*. have reported excellent results using HA to fill massive acetabular [63] and femoral defects [64] during revision hip surgery. This is despite the joint reaction force experienced by the hip joint following arthroplasty being in the range of 250%−360% of body weight [65]. Oonishi *et al*. observed that the two main disadvantages of HA when used *in vivo* were the difficulty of placement and retention in defects and the time taken for restoration of bone. Their work on animal models suggested that Bioglass® had the advantage of ease of handling and rapid resorption (two weeks c.f. 12 weeks for HA) [66]. Coralline HA grafts have also demonstrated acceptable results in the medium term for acetabular reconstruction in complex revision hip surgery [67].

In posterior spinal fusion for idiopathic scoliosis, Triosite, a synthetic porous ceramic (60% HA:40% TCP) demonstrated equivalent results in terms of fusion when compared to autograft. The ceramic group demonstrated a lower incidence of wound complications post operatively [28]. It was felt this difference was due to the lack of an antigenic response to the material, an observation supported by other authors [68].

A number of authors have reported the use of ceramic bone graft substitutes as a graft expander used in conjunction with autograft or allograft [62,69,70]. The results in revision hip surgery were good in the short to medium term. The relatively small sizes of these series and the mixed indications for revision as well as the different graft combinations limit the usefulness of the information available to date. A recent long term cohort study comparing the use of allograft alone and a 50:50 mix of HA and allograft in impaction grafting for revision total hip replacement showed promising survival rates that were not significantly different between the two groups at 13 years (84% allograft alone, 82% allograft/HA mixture) [71]. This study also demonstrated comparable graft incorporation and patient scores between both groups.

The problems with early massive subsidence in some autograft impaction grafting series [11] have not been demonstrated by other authors. Massive subsidence appears to be associated with more extensive femoral cortex destruction and defects in the initial stem-allograft construct created [72]. Other authors have identified that instability leading to massive subsidence is multifactorial and ultimately implant stability must be determined at the time of surgery [73]. Histological analysis of samples from the interface in clinically sound constructs has demonstrated bone remodelling and partial restoration of bone stock between 11 and 27 months [74]. Improved instrumentation has a role to play in increasing the success of impaction grafting procedures with vibration and drainage identified to be important in surrogate markers of reducing the risk of fracture and improving graft strength [75]. The use of coralline graft with bone marrow in isolation demonstrated poor results, when used in conjunction with autograft, the results were good [76]. The use of a hydroxyapatite-bioactive glass ceramic composite in isolation demonstrated significantly worse results than autograft alone in the context of posterolateral lumbar spine fusion [77].

It has been suggested that pharmacological agents may have a stimulatory effect on the resorption of the ceramic bone graft or bone ingrowth [78,79]. More new bone would appear to be formed with silica based bioactive glass used as a graft in an animal model when a bisphosphonate is given in conjunction, the amount is however less than if the bisphosphonate is given in isolation [79]. Bisphosphonates are highly selective for bone. Different types of bisphosphonate vary in their precise mechanism of action but broadly, the class of drugs leads to an inhibition of bone resorption. Mechanisms of influence on osteoclasts include inhibiting formation, recruitment, differentiation, resorption and inducement of apoptosis. The authors of the study theorised that the biospheres that they used induced a high rate of bone turnover, the addition of the bisphosphonate was felt to shift this turnover towards a positive balance of bone deposition by inhibiting the resorption. The only clinical failure of BoneSave (a porous biphasic ceramic) observed in our unit has been in a patient undergoing bisphosphonate therapy in the peri- and postoperative period. Statins would appear to have no effect when administered systemically and a detrimental effect when administered locally despite their osteoblastic and osteoclastic effects [78]. A beneficial effect in terms of bone ingrowth into the pores of HA ceramic has been demonstrated in rabbits when low-intensity pulsed ultrasound is applied without a detrimental effect on the mechanical strength of the ceramic [80].

## 3. Conclusions

The ideal bone graft substitute should exhibit a number of properties in order to allow its widespread use as a realistic and cost effective alternative to autograft or allograft. The material should:

1. Be inexpensive
2. Be available in unlimited quantities
3. Be reproducible
4. Provide structural stability at least until substituted by host bone
5. Allow neo-ossification by means of osseoconduction and osseoinduction
6. Have no infectivity
7. Provoke no antigenicity

The improvement in the available bone graft substitutes is leading to a decrease in the reliance on auto- and allograft for use in revision arthroplasty surgery. There are a growing number of clinical trials demonstrating good results in the short, medium and now long term. Further long term results in large series of patients are awaited with interest. The potential to combine these absorbable substitutes with pharmacological agents, mechanical stimulus or morphogens such as transforming growth factor beta, bone morphogenic proteins or cartilage derived morphogenic proteins creates the possibility of restoration of massive bone loss at revision surgery.
